# Amplitude-modulation detection by recreational-noise-exposed humans with near-normal hearing thresholds and its medium-term progression

**DOI:** 10.1016/j.heares.2014.09.005

**Published:** 2014-11

**Authors:** Michael A. Stone, Brian C.J. Moore

**Affiliations:** a Audiology and Deafness Group, School of Psychological Sciences, University of Manchester, Manchester, M13 9PL, UK; b Auditory Perception Group, Department of Experimental Psychology, University of Cambridge, Downing Street, Cambridge CB2 3EB, UK

**Keywords:** AM, amplitude modulation, DPOAE, distortion-product otoacoustic emission, HN, high-noise exposure, IHC, inner hair cell, LN, low-noise exposure, OHC, outer hair cell, PLL, preferred listening level, PMP, personal music player, psd, power spectral density, SL, sensation level, SOAE, spontaneous otoacoustic emission

## Abstract

Noise exposure can affect the functioning of cochlear inner and outer hair cells (IHC/OHC), leading to multiple perceptual changes. This work explored possible changes in detection of amplitude modulation (AM) at three Sensation Levels (SL) for carrier frequencies of 3, 4 and 6 kHz. There were two groups of participants, aged 19 to 24 (Young) and 26 to 35 (Older) years. All had near-normal audiometric thresholds. Participants self-assessed exposure to high-level noise in recreational settings. Each group was sub-grouped into low-noise (LN) or high-noise (HN) exposure. AM detection thresholds were worse for the HN than for the LN sub-group at the lowest SL, for the males only of the Young group and for both genders for the Older group, despite no significant difference in absolute threshold at 3 and 4 kHz between sub-groups. AM detection at the lowest SL, at both 3 and 4 kHz, generally improved with increasing age and increasing absolute threshold, consistent with a recruitment-like process. However, poorer AM detection was correlated with increasing exposure at 3 kHz in the Older group. It is suggested that high-level noise exposure produces both IHC- and OHC-related damage, the balance between the two varying across frequency. However, the use of AM detection offers poor sensitivity as a measure of the effects.

## Highlights

• Young males exhibit deficits in AM detection due to recreational noise exposure.
• Older participants of both genders exhibit a similar deficit due to noise exposure.
• Higher absolute thresholds were associated with better AM detection.
• Results are consistent with a combination of IHC and OHC dysfunction.
• AM detection at 3 kHz was correlated with cumulative noise exposure.

## 1 Introduction

Noise-induced hearing damage in humans is associated with an increase in absolute threshold in the frequency range 3–6 kHz, which can later spread to between 2 and 8 kHz (Smoorenburg, 1990). Above 8 kHz, audiometric thresholds may remain near-normal, but with prolonged exposure, the region between 12 and 20 kHz is also affected (Fausti et al., 1981, Hallmo et al., 1995). The earliest sign of damage often takes the form of a notch in the audiogram, centered between 3 and 6 kHz (Fowler, 1929, Coles et al., 2000). The notch can be quite narrow, and can be missed if audiometry is performed only at octave frequencies (West and Evans, 1990).

The audiogram is recognized as being an insensitive measure for quantifying hearing damage, since there may be changes in hearing even when audiometric thresholds are within the “normal” range. Such changes include tinnitus (Davis et al., 1950, Moore, 2012), broadening of the auditory filters (West and Evans, 1990), and reduced otoacoustic emissions (Attias et al., 1998, Hall and Lutman, 1999, Lucertini et al., 2002). Also, substantial loss of synapses and degeneration of neurons in the auditory nerve may occur without any marked effect on the audiometric threshold (Schuknecht, 1993, Kujawa and Liberman, 2009, Lin et al., 2011, Liu et al., 2012). Early detection of “sub-clinical” or “hidden” hearing losses has therefore attracted increasing attention, to allow identification of individuals who are at risk and to take steps to avoid further damage (Fausti et al., 1981, Lucertini et al., 2002, Stone et al., 2008, Schaette and McAlpine, 2011). The present paper describes a study using a perceptual measure, namely the detection of amplitude modulation (AM) applied to a low-level sinusoidal carrier, which might be useful in early identification of one manifestation of noise-induced hearing damage.

With increased regulation to limit noise levels and exposures arising in the workplace, the focus has moved to possible damage from noise exposure in “recreational” settings, which attract little or no regulation. Of particular concern are the risks from personal music players (PMPs) and amplified music events. In the UK, the prevalence of these contributions to noise exposure was estimated to have tripled between 1980 and 1994 (Smith et al., 2000). Although combinations of PMPs and headphones/earphones are capable of producing sound levels in the range 90–120 dBA (Fligor and Cox, 2004), preferred listening levels (PLL) are generally much lower than this (Kuras and Findlay, 1974, Bradley et al., 1987, Williams, 2005, Torre, 2008, Worthington et al., 2009, Shimokura and Soeta, 2012). The PLL found in such studies indicate that the exposures would be regarded as potentially injurious only with prolonged listening, as defined by the methods used in industrial regulation (ISO 1999, 1990), although PLLs and exposure durations can vary significantly with factors such as gender and ethnicity (Torre, 2008, Vogel et al., 2008). Exposures of shorter duration, but with the potential to cause damage, have been have been found to occur in night clubs or at rock concerts, where levels in excess of 95 dBA are regularly encountered (Sadhra et al., 2002, Bray et al., 2004, Santos et al., 2007, Stone et al., 2008, Potier et al., 2009). In some of these reports, mean exposures of around 110 dBA were not uncommon, but with durations not exceeding a few hours.

The pattern of sound-induced damage and its progression in humans is not easy to determine. The “equal energy” hypothesis, which forms the basis of workplace regulation, assumes that physiological damage is proportional to energy received (Ward et al., 1981). However, it has long been recognized that additional factors play a role, such as the frequency and duration of “recovery” periods and the degree of impulsiveness of the sound (Ward, 1970, Ward et al., 1981, Davis et al., 2009). Ward et al. (1981) also noted that, above a certain “critical” sound level, more damage was observed than predicted by the equal-energy hypothesis. Caution is needed when comparing animal to human data, since noise damage in animals is usually produced by narrowband continuous steady signals, while the sounds experienced by humans are usually broadband, and vary markedly in spectrum and in temporal pattern. Borg et al. (1995) proposed that, for humans, OHC damage resulted from prolonged exposures to moderately high intensities, whereas loss of IHC function was associated more with impact or very high-intensity sounds, implicitly implying that there was a critical level for humans.

The perceptual consequences of damage to the IHCs, loss of synapses, and degeneration of primary auditory neurons are likely to be similar, in that all would result in a reduced fidelity of coding of information in the auditory nerve. In what follows, dysfunction in IHCs/synapses/neurons is referred to as IHC dysfunction for brevity. Such dysfunction would be expected to lead to impaired performance in discrimination tasks, but to have little or no effect on absolute thresholds, since only a few functioning neurons are required for detection of a signal. Conversely, OHC damage would be expected to produce reduced gain of the cochlear amplifier (Robles and Ruggero, 2001), thereby elevating absolute thresholds, and to loss of cochlear compression, perhaps affecting the perception of loudness (Moore and Glasberg, 2004) and of envelope fluctuations (Moore et al., 1996).

A few studies have employed psychoacoustical measures in addition to the audiogram in attempts to detect early hearing damage in humans. Studies with animals (Kujawa and Liberman, 2009, Furman et al., 2013) suggest that noise exposure initially leads to neuropathy mainly for neurons with low spontaneous rates and medium to high thresholds. This leads to the prediction that perceptual deficits should be apparent at medium to high presentation levels. Consistent with this, Kumar et al. (2012) performed all of their testing in humans at 80 dB SPL, and identified deficits in some psychoacoustic and speech perception tasks for their noise-exposed group.

A disadvantage of presentation at high levels is that the stimuli produce excitation of the cochlea over a considerable portion of its length, and hence performance is based on the integrated outputs of many neurons. Evidence from humans (Stone et al., 2008, Vinay and Moore, 2010) suggests that some forms of sub-clinical damage may be localized on the cochlea, and their observable effects may therefore be diluted by use of signals producing widespread excitation. An alternative approach is to use narrowband stimuli presented at low sensation levels (SLs), so as to restrict stimulus-evoked excitation to a small region of the cochlea. Any perceptual effects measured with such stimuli are likely to reflect mechanisms separate from that identified by Kujawa and Liberman (2009) and Furman et al. (2013).

Vinay and Moore (2010) investigated the perceptual effects of PMP use for male participants who habitually listened at self-judged high replay levels (not measured) for at least 2 h per day. Male participants were used because of their tendency to use higher levels than females (Torre, 2008). Vinay and Moore reported that PMP users had better AM detection thresholds but poorer frequency discrimination thresholds than a control group, even though both groups had audiometric thresholds within the normal range. Vinay and Moore proposed that the better AM detection thresholds might be a result of OHC dysfunction, which leads to steeper input–output functions on the basilar membrane (Robles et al., 1986) and increases the perceived magnitude of amplitude fluctuations (Moore et al., 1996). They suggested that the poorer frequency discrimination thresholds could result from reduced frequency selectivity associated with OHC dysfunction, or reduced sensitivity to temporal fine structure, perhaps associated with IHC dysfunction (Moore, 2014), or both.

Stone et al. (2008) tested an experimental group comprising rock musicians and attendees at night clubs, who were regularly exposed to sound levels above 100 dBA (verified by a dosimeter), but only for a few hours per week. The task was to discriminate a Gaussian noise from a low-noise noise (Pumplin, 1985) with reduced envelope fluctuations. The experimental group performed more poorly than a non-exposed control group, even though both groups had audiometric thresholds within the normal range. This pattern was attributed to IHC dysfunction in the experimental group, which would have led to a “noisier” neural representation of the signal envelope.

Vinay and Moore (2010) found better AM detection for their exposed group, while Stone et al. (2008) found poorer envelope discrimination for their exposed group. The apparent discrepancy may have occurred because PMP use involves sub-critical levels, while attending rock/club events involves at least some super-critical levels, leading to IHC damage. However, the discrepancy may also be related to the difference in the tasks used (AM detection versus envelope discrimination) and in the center frequencies used (0.5, 3, 4, and 6 kHz for Vinay and Moore; 2, 3 and 4 kHz for Stone et al.). Also, both studies had the limitation that the control group had a 6–7 year greater mean age than the experimental group, and there was a large range of ages within all groups.

The present study was intended to explore the origin of the discrepancy between the results of the two studies discussed above, while controlling more precisely for the effects of age. Since the task used by Stone et al. (2008) required rather a lot of training to achieve stable results, the present study used an AM-detection task similar to that employed by Vinay and Moore (2010). The specific questions addressed were:

(1) Does AM-detection performance for groups who are probably exposed to super-critical levels depend on the amount of the exposure? To address this, two separate experimental groups were enrolled, with ages 19 to 24 and 26–35 years. The Older group had been exposed for a longer period than the Young group. Also, the amount of exposure varied within each group.
(2) Is AM-detection performance related to the function of the OHC, as assessed using otoacoustic emissions and absolute thresholds?

## 2 Materials and methods

### 2.1 Participants and their recruitment

We wished to assess the effects of exposure intensity and duration on AM detection. Ideally, this would be done using a longitudinal study. However, such studies are time consuming. An alternative approach is to recruit separate groups, differing in age and duration of noise exposure, and to compare results across groups. This cross-sectional approach was adopted here. Two groups of participants were recruited on the basis of age. In order to reduce the influence of demographic factors, they were recruited from a population of university undergraduates and graduates.

Each potential participant completed a questionnaire, the results of which were used to exclude people who had suffered head traumas, repeated ear surgery, neurological problems or chronic ear infections. The questionnaire also requested estimates of weekly noise exposure to PMP and to high-noise events such as nightclubs or rock concerts: this section of the questionnaire is included in the Appendix. Folk or jazz concerts were not considered as “high-noise” events. Questions included the participant's age of first exposure in either category and the pattern and duration of exposure during their 3–4 year period as undergraduates and in 5-year blocks thereafter up to their chronological age. Participants were excluded if the questionnaire revealed that they were regularly exposed to high-level sounds from other sources, such as motor sports or farm machinery. The 43 participants in the “Young” group had a mean age of 21 years (standard deviation, sd = 1.4 years, range 18–24 years). The 36 participants in the “Older” group had a mean age of 29 years (sd = 2.6 years, range 26 to 35 years). The Young group was recruited 2 years before the Older group. The Older group, comprising mostly graduates, was more international in origin than the Young group, comprising mostly undergraduates.

Makary et al. (2011) showed that counts of spiral ganglion cells (SGC) in human temporal bones decline with increasing age, seemingly independent of noise exposure sufficient to cause cellular damage. According to their study:

(1) $${\text{SGC}}_{\text{count}}=32913-100.25\times \text{Age}$$

where SGC_count_ is the total count of SGCs in the cochlea and *Age* is expressed in years. The difference in mean age between the two groups used here would be expected to lead to a reduction in SGC_count_ for the Older group, which might have a confounding effect. However, according to Eq. (1), the ratio of SGC_count_ values for the two groups would be 1.026, i.e. very close to one. It seems likely, therefore, that differences in SGC_count_ would have only a very small influence on the results.

All participants underwent a battery of screening tests. Air-conduction manual audiometry was performed at frequencies between 125 and 8000 Hz, including 3000 and 6000 Hz. For the Young group, participants were rejected if any of their thresholds exceeded 15 dB HL in either ear. For the Older group, a laxer criterion was necessary in order to retain a group of sufficient size: participants were rejected if their thresholds:

(a) exceeded 20 dB HL at any frequency between 125 and 2000 Hz, inclusive, or
(b) exceeded 25 dB HL at the frequencies of 3000, 4000 or 6000 Hz.

One participant in the Older group had a threshold at 8 kHz of 35 dB HL, but all others had thresholds at 8 kHz ≤ 20 dB HL.

Using an Otodynamics “Otoread” handheld device, the levels of DPOAEs were recorded for all participants at 2, 3, 4 and 6 kHz. The primary tones had a frequency ratio of 1.2 and had levels of 65 and 55 dB SPL for the lower and upper frequency component, respectively.

The ear chosen for testing was assigned randomly to each participant in each group, unless the screening questionnaire disqualified the selected ear, or the audiogram revealed an asymmetry, in which case the better ear was selected.

The Older group was also tested to identify the frequency and level of possible spontaneous otoacoustic emissions (SOAEs) using an Etymotic Research ER10C probe microphone system, with recordings made to a high-quality solid-state recorder (Edirol R-44, 24 bit, 44.1 kHz sampling rate). (The SOAE recording system was not available at the time of testing of the Young group). Two separate recordings were made sequentially from each ear tested. Each recording lasted about 30 s. SOAEs were analyzed off-line using custom MATLAB scripts. Each recording was band-pass filtered between 1800 and 6900 Hz, and split into frames of 0.38 s duration, each overlapping 50% with the previous frame. The logarithmic mean power of all frames was measured, and frames with levels more than two sds below or one sd above the mean level were rejected. This acted to reject high-level extraneous sounds and also ensured that low-level portions of the recordings at onset and offset produced by the digital file manipulations were ignored. The mean power spectral density (psd) was calculated from the remaining frames. The mean and sd of the level around each carrier frequency used for the AM detection task (3, 4 and 6 kHz) were calculated by averaging across 20 “bins” in the psd. Our intention was to exclude a participant if an SOAE frequency was (a) within 100 Hz of one of the experimental test frequencies, (b) its level exceeded the mean noise level plus 3 sds and (c) the SOAE level was above −20 dB SL. This combination of criteria was intended to avoid an external carrier tone beating with an SOAE, thereby affecting AM detection (Long, 1993). In practice, no potential participants were excluded based on this criterion.

### 2.2 Masking noise

A masking noise was used to limit the audible excitation produced by the carriers used in the AM-detection experiment (3, 4, and 6 kHz). The noise spectrum was shaped so as to achieve the following goals, using the method for calculating excitation patterns described by Moore et al. (1997): the excitation pattern should have a broad flat region between 40 and 12,000 Hz; and a second flat region from 2400 to 7500 Hz should be superimposed on the first region, but producing a 30-dB higher excitation level. To avoid possible edge tones associated with sharp spectral edges (Fastl, 1971), the transitions between the two regions were shaped by a raised-sinusoid between 1900 and 2400 Hz and between 7500 and 9000 Hz. The spectrum of the noise allowed for the deviation of the headphone response from the diffuse-field response assumed in the excitation-pattern software. The spectrum was inverse Fourier transformed (assuming a random phase for each component) to produce a noise waveform of 24 s duration without repetition (2^20^ samples at 44,100 Hz sampling rate). The noise level, specified in a 1-ERB_N_-wide band (Glasberg and Moore, 1990) around the signal carrier frequency, was 20 dB below the level of signal. This level was chosen so as to limit the audible range of the excitation evoked by the signal. For signals presented at 10 dB SL, the masking noise was usually inaudible, and participants were warned not to be disturbed by this.

### 2.3 Experimental method and stimuli

Testing took place immediately after the audiometric screening and continued into a second session held on a separate day. Each session lasted up to 2 h. Each participant was allocated a random permutation of testing order for the three test frequencies, with the first frequency re-tested at the end. Data from the first center frequency to be tested were regarded as practice and were discarded.

For each test frequency, the absolute threshold was measured using an adaptive 2-alternative forced-choice method, with a 3-down, 1-up procedure tracking the 79%-correct point on the psychometric function (Levitt, 1971). Stimulus intervals were marked by lights on a screen, and visual feedback was provided. The step size was 5 dB until the first reversal, decreased to 3 dB until the next reversal, and then kept at 2 dB. Six reversals were obtained using the smallest step size and threshold was estimated as the mean level at the last six reversals. Two estimates were obtained for each frequency. If the mean of the two differed by more than 4 dB, or the sd of the reversal points for either estimate exceeded 4 dB, a further estimate was obtained. Absolute thresholds were calculated as the mean of all estimates. Thresholds were obtained in dB SPL and converted to dB HL using the measured response of the test headphones (HDA200) in the Zwislocki coupler of a KEMAR manikin, and the values of monaural minimum audible pressure (MAP) calculated using the model of Moore and Glasberg (2007).

Once an absolute threshold had been determined, AM detection thresholds were measured for signals with carriers presented at 10, 25 or 40 dB SL. The same 2-alternative forced-choice, 3-down, 1-up procedure was used. One interval contained an unmodulated carrier at the desired center frequency, while the other interval contained the same carrier with AM at a rate of 25 Hz. The choice of an AM rate of 25 Hz was motivated by three factors:

(1) The spectral sidebands produced by the AM would fall within the passband of the auditory filter centered at the carrier frequency for all values of the carrier frequency.
(2) The rate lies well within the region of maximum sensitivity in the temporal modulation transfer function for humans (Kohlrausch et al., 2000).
(3) AM detection improves with increasing number of modulation cycles up to some limit (Sheft and Yost, 1990). The use of a 25-Hz AM rate meant that many modulation cycles occurred with a reasonably short stimulus, avoiding the need for long presentation intervals and reducing fatigue and boredom.

The RMS level of the modulated stimulus was adjusted to match that of the unmodulated stimulus, independent of the modulation depth *m*. The starting value of *m* was 0.4467, equivalent to −7 dB when expressed as 20log_10_(*m*). The step size in *m* was 4 dB until one reversal occurred, after which it decreased to 2 dB until the next reversal, when it decreased to 1 dB. Six reversals were obtained at the smallest step size and the threshold estimate was taken as the mean value of 20log_10_(*m*) at the last six reversals. Presentation order was randomized across SL, and two estimates were obtained for each SL.

Stimuli were 250 ms in duration, including 10-ms raised-cosine ramps, and were separated by a gap of 400 ms. The stimuli were presented in a sample of the masking noise drawn randomly on every trial from within the 24-sec duration file. The noise started 200 ms before the first stimulus and ended 200 ms after the end of the second stimulus in a trial. The noise sample was ramped on and off with 25-msec raised-cosine ramps.

### 2.4 Signal generation and presentation

Stimuli were generated with 24-bit precision using a 44.1-kHz sample rate, converted to analog form using a LynxONE soundcard (Young group) or a Lynx L22 soundcard (Older group), and routed through a Mackie 1202 VLZ-PRO mixing desk. Stimuli were presented via Sennheiser HDA200 circumaural headphones. Since the sensitivity of the HDA200 headphone is high, and only low-level signals were required, the stimuli were passively attenuated by 42 dB just prior to the headphone cable to ensure that electrical system noise was inaudible. The participant was seated in a double-walled sound-attenuating chamber.

Once the headphones were comfortably positioned, participants were instructed not to move or touch them, apart from in an emergency, until all the measures for that particular center frequency had been gathered. This was intended to reduce possible variation of level with headphone placement. The headphone cable was clipped to clothing near the neck of the participant, leaving a small loop for unrestricted head movements. This reduced the transmission of movement noises via the cable to the earpiece, which could have been a distraction during the presentation of low-level signals.

## 3 Results

### 3.1 Self-reported noise exposure and division into sub-groups

On the basis of the questionnaire responses, we estimated the cumulative amount of exposure to recreational events at which sound levels were probably 100 dBA SPL or more (hence primarily derived from nightclubs or rock concerts). The measure was based on the average number of hours per week for which such high-level exposure occurred, *H*, multiplied by the number of years, *N*, over which such weekly exposure occurred.

**Fig. 1:**
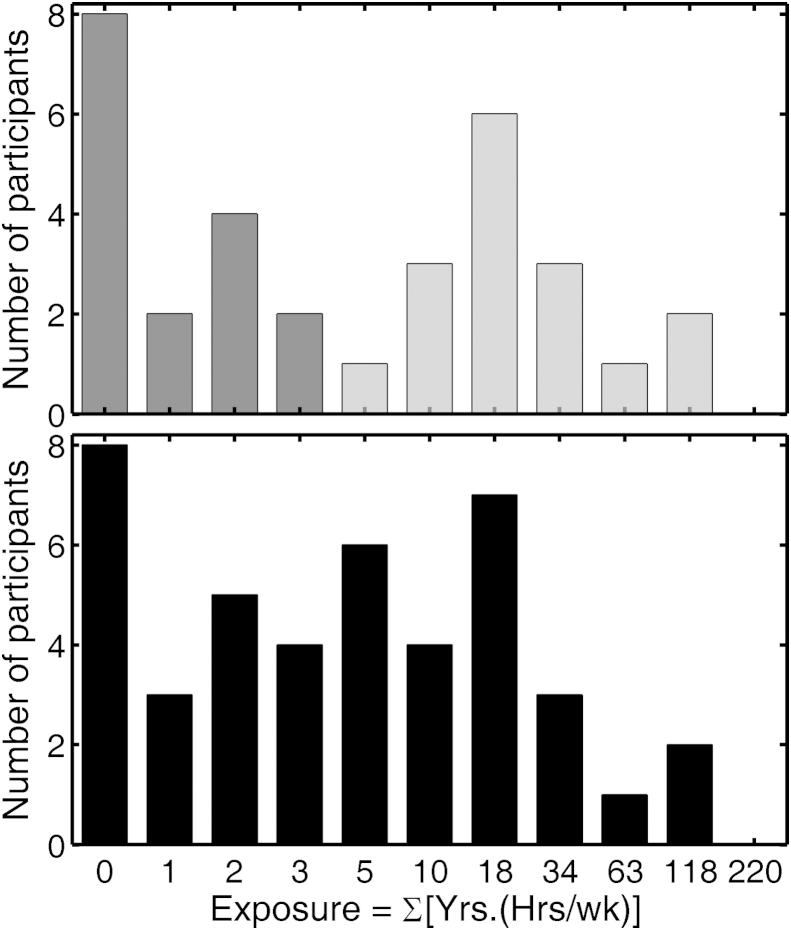
Histograms of measures of self-reported exposure for the Young group. The bottom panel is for the full 43-member group. The top panel is for 32 participants selected to have low-noise (LN, dark gray) or high-noise (HN, light gray) exposure. “Bin” centers are logarithmically spaced. Abscissa labels are given to the nearest integer.

A histogram of the exposure values for the Young group showed a distinct skewness when linearly-spaced bins were used. Hence, for plotting and analysis purposes, the centers of the bins were spaced on a logarithmic scale, resulting in a more normal-shaped distribution. This scale transformation reduces the bias that might otherwise occur in regressions due to the presence of “outliers”. The lower panel of Fig. 1 shows histograms of the calculated exposures for the 43 members of the Young group. The numbers on the abscissa have been rounded to the nearest integer. The bin labeled “0” includes five participants who had never been exposed to any high-noise events and three participants with exposure values less than unity. A sub-group of 32 participants was chosen whose histogram of exposures could be divided into two non-overlapping groups and whose group-mean exposures were widely separated. The histogram for the 32 participants is shown in the upper panel of Fig. 1, where the low-noise (LN) sub-group is indicated by dark gray shading and the high-noise (HN) sub-group by light gray shading. The LN and HN groups were equal in size and were gender-balanced. There were equal numbers of left and right ears in each sub-group for each gender and amount of noise exposure. This selection enabled a multi-factorial Analysis of Variance (ANOVA) to be performed for each of the outcome measures. The ANOVAs for measures of absolute threshold and DPOAE had between-subject factors of exposure (LN/HN), gender (male/female), and ear (left/right) and within-subject factors of frequency (3, 4 and 6 kHz). For the ANOVA of AM detection thresholds, there was an additional within-subject factor of presentation level (10, 25 and 40 dB SL).

**Fig. 2:**
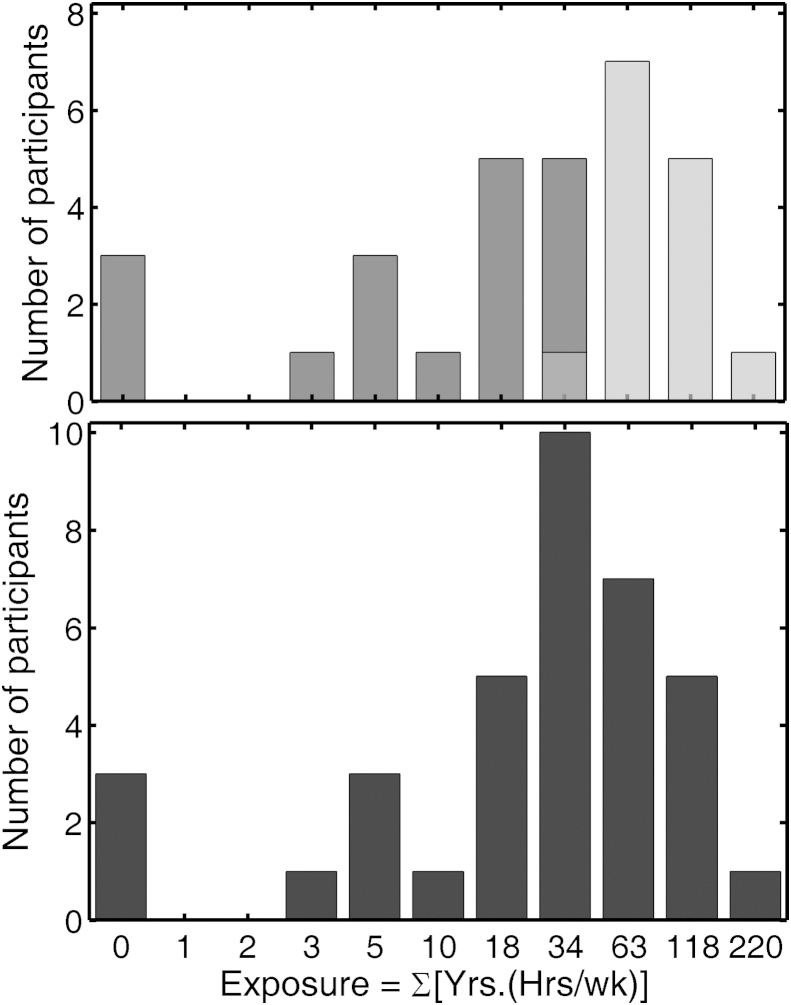
Histograms of measures of self-reported exposure for the Older group. The bottom panel is for the full 36-member group. The top panel is for 32 participants selected to have LN (dark gray) or HN (light gray) exposure. “Bin” centers are logarithmically spaced, with the same center values as used for Fig. 1. Abscissa labels are given to the nearest integer.

For the Older group, the histogram of exposure values was again skewed when linearly spaced bins were used. To permit direct graphical comparison with the histograms for the Young group shown in Fig. 1, the same logarithmic transform was used. The resulting histogram for the 36 participants in the Older group is shown in the lower panel of Fig. 2. The bin labeled “0” includes two participants who had never been exposed to any high-noise events and one with an exposure value less than unity. It is clear that the distribution has markedly higher mean and median values than for the Young group. To produce two groups separated by degree of exposure, a sub-group of 32 participants was selected to include those with either a LN (below 38) or HN (above 38) exposure. The histograms for the two sub-groups are indicated in Fig. 2 by dark gray shading and light gray shading, respectively. The LN and HN sub-groups were less clearly separated than for the Young group. Bin “34” included one participant for the HN group with an exposure value of 38.5 and two from the LN group with an exposure value of 37. The sub-groups included 18 LN (8M, 10F) and 14 HN (9M, 5F) participants. Hence, it was not possible to maintain statistical balance in the factor gender (and also factor ear) between the two sub-groups, so in ANOVAs of the data for the Older group, these factors were not considered.

### 3.2 ANOVAs for Young group

**Fig. 3:**
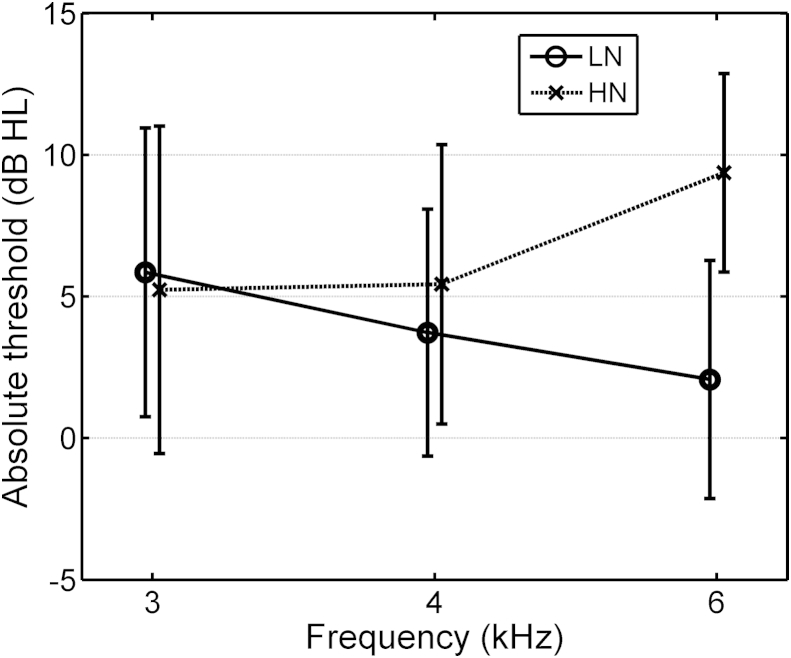
Mean absolute thresholds at 3, 4 and 6 kHz for the LN- and HN-exposed sub-groups of the 32-member Young group. Error bars show ±1 standard deviation (sd).

The ANOVA for absolute threshold (Table 1(1)) yielded significant effects of Exposure, and significant interactions of Exposure × Frequency and Gender × Frequency. The interaction of Exposure × Frequency is shown in Fig. 3. Mean absolute thresholds were similar for the LN and HN groups at 3 and 4 kHz, but were 7.3 dB lower (better) for the LN group than for the HN group at 6 kHz.

**Table 1:** Significant effects from the ANOVA for the sub-group of 32 Young participants. Factors were Exposure (LN, HN), Frequency (3, 4 or 6 kHz), Gender (male/female), Ear (left/right), and for AM detection, sensation level (10, 25 and 40 dB SL).

Factor	df	F Ratio	Probability	Description of effect
**(1) Absolute threshold**				
Exposure	1,24	4.42	0.046	2.8 dB higher thresholds for HN group.
Exposure.Frequency	2,48	12.6	<0.001	HN exposure increases thresholds only at 6 kHz; see Fig. 3.
Gender.Frequency	2,48	4.00	0.025	Females more sensitive at 4 kHz than males.
**(2) Level of DPOAEs**				
Frequency	2,48	16.6	<0.001	Recorded levels decrease with increasing frequency.
Exposure.Frequency.Ear	2,48	3.81	0.029	HN group always have lower DPOAEs than LN group. Within each group, DPOAE levels are similar between ears at 3 and 4 kHz. At 6 kHz, left DPOAE level lower than right for LN group, but reverse pattern for HN group.
**(3) AM detection thresholds**				
Frequency	2,48	19.6	<0.001	Thresholds worse at 6 kHz than at 3 and 4 kHz.
SL	2,48	40.3	<0.001	Thresholds worst at 10 dB SL, best at 25 dB SL.
Frequency.SL	4,96	7.53	<0.001	Threshold at 3 kHz same at 25 and 40 dB SL, differing from pattern of SL described above.
Gender.Frequency	2,48	5.88	0.005	Males better than females at 4 and 6 but not 3 kHz.
Exposure.Gender.SL	2,48	3.79	0.030	HN males worse at 10 dB SL than LN males (see Fig 4, left panel).
Exposure.Gender.Frequency	2,48	3.85	0.028	For females, AM detection changes more with frequency for LN group than for HN group. (see Fig 4, right panel).

The ANOVA for DPOAE levels (Table 1(2)), showed a significant effect of frequency. DPOAE levels generally decreased with increasing frequency, possibly because reverse transmission of DPOAEs through the middle ear is less efficient at high frequencies. We do not understand the three-way interaction of Exposure, Frequency and Ear, but it accounted for only a small proportion of the variance in the data.

**Fig. 4:**
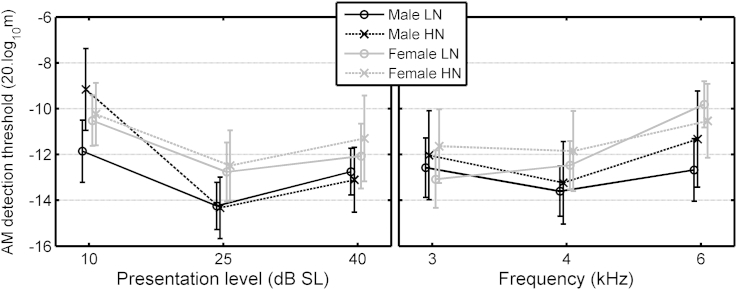
Mean AM detection thresholds for the Young group, as a function of presentation level (averaged across frequency, left panel) or carrier frequency (averaged across presentation level, right panel), with the LN and HN groups separated by gender. Error bars show ±1 standard error (se).

The ANOVA for AM-detection thresholds (Table 1(3)) gave two significant interactions involving Exposure. The three-way interaction of Exposure, Gender and SL is illustrated in the left panel of Fig 4. The HN males (black crosses) had poorer AM-detection thresholds at 10 dB SL than the LN males; *t*(48) = 3.98, *p* < 0.001 (2-tailed). The three-way interaction of Exposure, Gender and Frequency is illustrated in the right panel of Fig 4. AM detection thresholds varied more across frequency for the LN females (gray circles) than for the HN females or for the males. Again, this interaction is hard to interpret, but it accounted for only a small proportion of the variance in the data.

### 3.3 ANOVAs for Older group

The ANOVA for absolute threshold (Table 2(1)) gave a significant effect of frequency; the mean threshold was 4.1 dB higher at 3 than at 4 and 6 kHz. The ANOVA for DPOAE levels (Table 2(2)), showed a significant effect of frequency. Again, DPOAE levels generally decreased with increasing frequency.

**Table 2:** Significant effects from the ANOVA for the sub-group of 32 Older participants. *N.S*. denotes “not significant”.

Factor	df	F Ratio	Probability	Description of effect
**(1) Absolute threshold**				
Frequency	2,60	5.32	0.007	Mean thresholds 4.1 dB worse at 3 kHz than at 4 and 6 kHz.
**(2) Level of DPOAEs**				
Frequency	2,60	27.1	<0.001	Recorded levels decrease with increasing frequency.
**(3) AM detection thresholds**				
Frequency	2,60	20.2	<0.001	Thresholds worse at 6 kHz than at 3 and 4 kHz.
SL	2,60	15.5	<0.001	Thresholds worst at 10 dB SL, similar at 25 and 40 dB SL
Frequency.SL	4,120	7.51	<0.001	Threshold at 6 kHz is generally higher (worse) than at 3 and 4 kHz but threshold at 40 dB SL is especially poor.
Exposure.SL	2,60	3.66	0.032	HN-exposed group have higher thresholds at 10 dB SL than LN group; see Fig. 5, left panel.
Exposure.Frequency.SL	4,120	3.20	0.016	Same pattern as for Exposure.SL, but only at 3 and 4 kHz, not 6 kHz. See Fig. 5, right panel.
**(3.1) AM detection thresholds separately for each frequency**				
3 kHz: Exposure.SL	2,60	7.13	0.002	HN group have higher thresholds than LN group at 10 dB SL, but similar thresholds at 25 and 40 dB SL. Compare black lines in Fig. 5, right panel.
4 kHz: Exposure.SL	2,60	4.08	0.022	HN group have higher thresholds than LN group at 10 dB SL, but similar thresholds at 25 and 40 dB SL. Compare mid-gray lines in Fig. 5, right panel.
6 kHz: Exposure.SL	2,60	0.10	*N.S.*	No difference between LN and HN groups. Compare light-gray lines in Fig. 5, right panel.

**Fig. 5:**
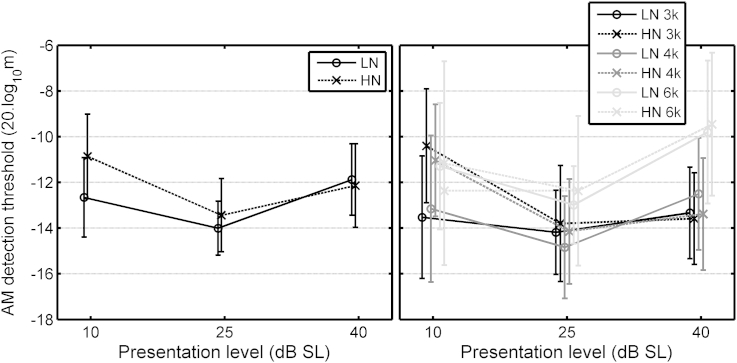
Mean AM detection thresholds for the LN and HN sub-groups of the Older group, plotted as a function of presentation level. The left panel shows the results averaged across the three carrier frequencies, 3, 4 and 6 kHz. The right-hand panel shows the same data but separated by carrier frequency. Error bars show ±1 se in the left panel and ±1 sd in the right panel.

The ANOVA for AM detection thresholds (Table 2(3)) gave two significant interactions involving exposure. The interaction of Exposure and SL is illustrated in the left panel of Fig. 5. AM detection thresholds were similar for the LN and HN groups at 25 and 40 dB SL, but mean threshold was higher for the HN group at 10 dB SL. The interaction of Exposure × Frequency × SL is illustrated in the right panel of Fig. 5. At 10 dB SL, AM detection thresholds were higher for the HN than for the LN group at 3 and 4 kHz, but not at 6 kHz.

This pattern of results was confirmed by ANOVAs conducted separately for each frequency. The outcomes are shown in Table 2(4). The interaction of Exposure × SL was significant at 3 and 4 kHz, but not at 6 kHz.

In summary, the results show an effect of Exposure on AM detection, but only for the frequencies of 3 and 4 kHz at 10 dB SL.

### 3.4 ANOVAs of combined data from Young and Older participants

A final series of ANOVAs was performed on the data sets combined, with Age group (Young/Older) and Exposure (LN/HN) as between-subject factors. The Young and Older groups each comprised 32 participants, and each Age group was divided into LN and HN sub-groups, as described earlier.

One problem in interpreting the ANOVA results of the combined data sets is that the effects of Age and Exposure (a cumulative measure) are partially confounded. The ANOVAs reported below keep the same LN/HN groupings as for the separate analyses, but here the LN/HN distinction should be interpreted more as a factor reflecting lifestyle than a factor reflecting cumulative exposure. To assess whether Age and Exposure were at least partially independent factors, we calculated correlations between the main data variables of the combined data sets while partialling out the effects either of Exposure or of Age. Data from participants with zero exposure were excluded. All values of Exposure were transformed onto a logarithmic scale. When the effect of Age was partialled out, there was a significant *positive* correlation of Exposure with AM detection threshold at 3 kHz and 10 dB SL (**r**_**72**_ = 0.280, *p* = 0.018). When the effect of Exposure was partialled out, there was a significant *negative* correlation of Age with AM detection at 3 kHz and 10 dB SL (**r**_**72**_ = −0.376, *p* = 0.001). This suggests that Age and Exposure have at least partially independent effects.

**Fig. 6:**
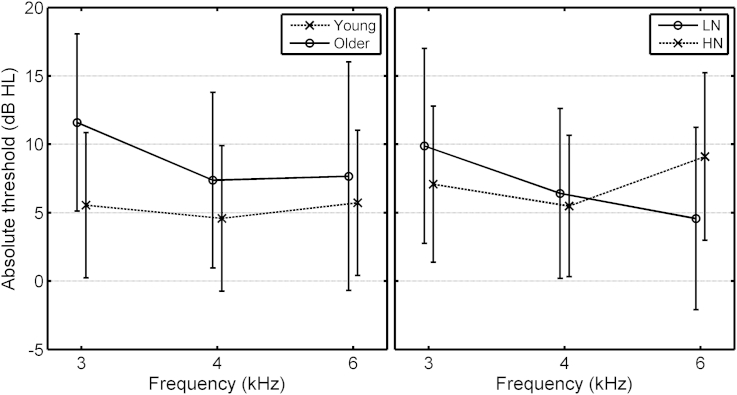
Mean absolute thresholds at 3, 4 and 6 kHz for the Young and Older age groups (left panel) and for the LN and HN groups (combined across Young and Older groups, right panel). Error bars show ±1 sd.

For Absolute Threshold (Table 3(1)), there was a significant effect of Age group, *F*(1,60) = 9.72, *p* = 0.003, reflecting an increase in three-frequency average threshold from 5.3 dB HL for the Young group to 8.9 dB HL for the Older group. There was a significant effect of Frequency, the mean threshold being highest at 3 kHz and lowest at 4 kHz. There was a significant interaction of Age group × Frequency, which is illustrated in the left panel of Fig. 6. The effect of Age group was larger at 3 than at 4 or 6 kHz. There was a significant interaction of Exposure × Frequency, which is illustrated in the right panel of Fig. 6. The HN group had higher thresholds than the LN group only at 6 kHz. Thus, the results for Age group and for Exposure are somewhat inconsistent; for Age group, the largest effect was at 3 kHz, while for Exposure, the largest effect was at 6 kHz.

**Table 3:** Significant effects from ANOVAs with age group (Young and Older) as a factor.

Factor	df	F Ratio	Probability	Description of effect
**(1) Absolute threshold**				
Age	1,60	9.71	0.003	Older have thresholds 3.6 dB higher than Young.
Frequency	2,120	5.03	0.008	Thresholds non-monotonic with frequency, 3 > (6 = = 4) kHz.
Age.Frequency	2,120	3.28	0.041	Older group have higher thresholds than Young, especially at 3 kHz; see Fig. 6, left panel.
Exposure.Frequency	2,120	9.56	<0.001	Compared to LN group, HN group have 2.5 dB lower thresholds at 3 kHz, but 4.7 dB higher at 6 kHz; see Fig. 6, right panel.
**(2) Level of DPOAEs**				
Age	1,60	6.47	0.014	Older have lower levels than Young.
Frequency	2,60	42.6	< 0.001	Levels decrease with increasing frequency.
**(3) AM detection thresholds**				
Frequency	2,120	35.4	<0.001	Thresholds highest at 6 kHz, equal at 3 and 4 kHz.
SL	2,120	43.6	<0.001	Threshold highest at 10 dB SL, lowest at 25 dB SL.
Frequency.SL	4,240	14.77	<0.001	Threshold at 6 kHz is generally higher (worse) than at 3 and 4 kHz, but threshold at 40 dB SL is especially poor.
Age.SL	2,120	5.61	0.005	LN and HN groups similar at 25 and 40 dB SL, but Young worse than Older at 10 dB SL; see Fig. 7, left panel.
Exposure.SL	2,120	5.49	0.005	Young and Older groups similar at 25 and 40 dB SL, but HN worse than LN at 10 dB SL. See Fig. 7, right panel.

For DPOAEs (Table 3(2)), as anticipated, there was a significant effect of Age. The mean DPOAE levels at 3, 4 and 6 kHz were 3.8 dB lower for the Older group than for the Young group.

**Fig. 7:**
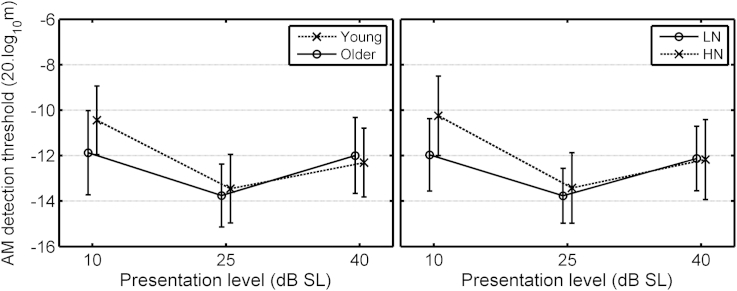
Mean AM detection thresholds at 3, 4 and 6 kHz for the Young and Older age groups (left panel) and for the LN and HN groups (combined across Young and Older groups, right panel). Error bars show ±1 se.

For AM detection (Table 3(3)), there was a significant effect of Frequency, the threshold being higher at 6 than at 3 or 4 kHz. There was a significant effect of SL, threshold being worst at 10 dB SL and best at 25 dB SL. There was a significant interaction of Age group and SL, which is illustrated in the left panel of Fig. 7. Threshold was lower (better) for the Older group than for the Young group at 10 dB SL, but not at the two higher SLs. There was a significant interaction of Exposure and SL, which is illustrated in the right panel of Fig. 7. Threshold was lower for the LN group than for the HN group at 10 dB SL, but not at the two higher SLs. Thus, the results appear somewhat paradoxical: at 10 dB SL, the Older group showed better AM detection than the Young group, despite the greater average exposure of the former, while the HN group showed poorer AM detection than the LN group. This may indicate two different underlying consequences of noise exposure, one operating over relatively long time scales that leads to better AM detection in the Older than in the Young group, and one operating over shorter time scales that leads to worse AM detection in the HN than in the LN group. These two processes could reflect OHC and IHC dysfunction, respectively.

### 3.5 Correlation analyses

In this section we describe correlations between the various measures. This was done separately for the two age groups, using the full data sets for each group. Note that gender and ear were not balanced in these groups. For the Young group, the logarithmic transform of the exposure values, as used for the histograms of Fig. 1, was also used in the correlation analyses. For the Older group, since the logarithmic transform of the exposure values, as used in Fig. 2, produced a reverse skew, the cube-root transform of the exposure values was also used in the correlation analyses. This transform effectively compressed the distribution of exposures for values less than 34. All probabilities associated with correlations, **r**_***N***_, are reported as two-tailed values, with (*N*−2) degrees of freedom, where *N* is the number of data points included in the calculation. Sections (1), (2) and (3) of Table 4 show the correlations between selected variables for the Young, Old, and Young and Old data sets combined, respectively. The correlations were between the absolute threshold, level of DPOAE, and AM detection threshold at 10 dB SL, at 3, 4 and 6 kHz. Section (3) includes an extra row of correlations with the level of the DPOAE recorded at 2 kHz (“OAE2k”). For the Young data set, OAE2k was correlated only with DPOAE level at 3 kHz (*p* < 0.001), while for the Older data set, OAE2k was correlated with the DPOAE level at both 3 and 4 kHz (both *p* < 0.001). Therefore, for brevity, OAE2k has been omitted from Table 4(1) and 4(2).

**Table 4:** Correlation tables for selected aspects of the data for the Young (Section 1), Older (Section 2), and the Young and Older groups combined (Section 3). Absolute thresholds at 3, 4 and 6 kHz are denoted by HL3k, HL4k and HL6k, respectively. Levels of DPOAEs at the same frequencies are denoted by OAE3k, OAE4k and OAE6k, respectively. AM detection thresholds for a presentation level of 10 dB SL at the same frequencies are denoted by the symbols AM3k, AM4k and AM6k, respectively. Double asterisks, “**”, denote a significant correlation at *p* < 0.001. A single asterisk, “*”, denotes a significant correlation at 0.01 > *p* ≥ 0.001. The hash symbol, “#”, denotes a significant correlation at 0.05 > *p* ≥ 0.01. N denotes the number of data points used in correlations. For the combined data set, the table includes an additional parameter, the level of the DPOAE at 2 kHz (OAE2k). See text for further details.

(1) Young participants, *N* = 43										
HL3k	–									
HL4k	0.517**	–								
HL6k	0.147	0.357#	–							
OAE3k	0.070	−0.099	−0.110	–						
OAE4k	−0.213	−0.378#	−0.214	0.539**	–					
OAE6k	−0.061	−0.259	−0.259	0.234	0.496**	–				
AM3k	−0.248	0.105	0.027	−0.206	−0.099	−0.181	–			
AM4k	0.015	0.120	−0.048	−0.130	−0.058	0.023	0.438*	–		
AM6k	0.009	0.258	−0.122	0.288	−0.032	−0.033	0.392*	0.567**	–	
	HL3k	HL4k	HL6k	OAE3k	OAE4k	OAE6k	AM3k	AM4k	AM6k	
(2) Older participants, *N* = 36										
HL3k	–									
HL4k	0.624**	–								
HL6k	0.133	0.182	–							
OAE3k	−0.284	−0.450*	0.063	–						
OAE4k	−0.179	−0.351#	0.047	0.691**	–					
OAE6k	−0.018	−0.242	−0.334#	0.421#	0.526**	–				
AM3k	−0.585**	−0.484*	−0.013	0.313	0.301	0.088	–			
AM4k	−0.426*	−0.534**	0.152	0.204	0.154	−0.082	0.463*	–		
AM6k	0.050	−0.263	−0.1400	0.066	−0.103	−0.214	0.291	0.267	–	
	HL3k	HL4k	HL6k	OAE3k	OAE4k	OAE6k	AM3k	AM4k	AM6k	
(3) Young and Older participants combined, *N* = 79										
HL3k	–									
HL4k	0.624**	–								
HL6k	0.187	0.270#	–							
OAE2k	−0.057	−0.169	0.086	–						
OAE3k	−0.252#	−0.363*	−0.037	0.667**	–					
OAE4k	−0.295*	−0.408**	−0.078	0.432**	0.658**	–				
OAE6k	−0.159	−0.298*	−0.317*	0.222#	0.373**	0.537**	–			
AM3k	−0.498**	−0.279#	−0.035	0.103	0.138	0.191	0.021	–		
AM4k	−0.293*	−0.305*	0.053	0.071	0.102	0.110	0.011	0.469**	–	
AM6k	−0.050	−0.080	−0.150	0.171	0.198	−0.026	−0.074	0.369**	0.418**	–
	HL3k	HL4k	HL6k	OAE2k	OAE3k	OAE4k	OAE6k	AM3k	AM4k	AM6k

Since there were some zero-valued self-reports of noise exposure, when calculating correlations with exposure values these data sets were excluded, to avoid biasing the correlations by a clustering effect. In what follows, only significant highlights from the tables of the correlations are discussed.

For the Young group, there was a significant correlation between absolute threshold at 6 kHz and exposure, **r**_**38**_ = 0.427, *p* = 0.008. There were no significant correlations between exposure and any of the measures of AM detection thresholds. There was only one other correlation of note, between DPOAE levels and absolute threshold at 4 kHz, **r**_**43**_ = −0.378, *p* = 0.013.

**Fig. 8:**
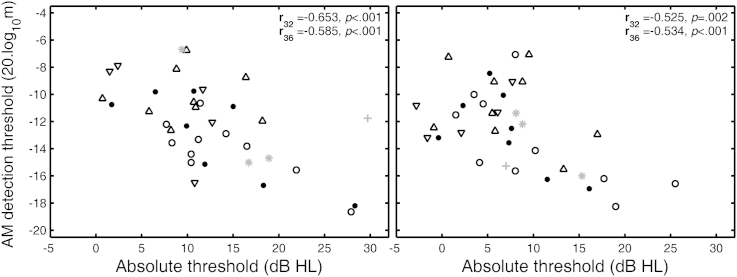
Scatter plots of AM detection thresholds at 10 dB SL against absolute thresholds for frequencies of 3 kHz (left) and 4 kHz (right). Data are for the 36-member Older group. In both panels, the reduced data set used in the ANOVAs is plotted with black symbols. Dots and circles show data for male and female members of the LN sub-group, respectively. Downward and upward pointing triangles show results for male and female members of the HN sub-group respectively. Gray “star” and “plus” symbols show data for male and female members of the full data set, respectively. **r**_**32**_ and **r**_**36**_ in the upper-right corner of each panel denote the correlations for the 32 members of the ANOVA data set or the full data set, respectively.

For the Older group, for the SL of 10 dB, there were significant correlations between absolute threshold and AM detection threshold at 3 and 4 kHz, but not at 6 kHz. Scatter plots for the frequencies of 3 and 4 kHz are shown in the left and right panels of Fig. 8. Both panels show a trend for improvement in AM detection threshold with increasing absolute threshold. This can be explained if increasing absolute threshold is associated with a recruitment-like process that increases the perceived modulation depth (Moore et al., 1996).

**Fig. 9:**
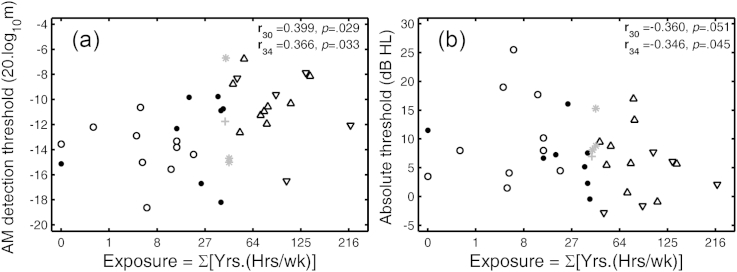
Scatter plots of (a) AM detection thresholds at 10 dB SL against exposure for a carrier frequency of 3 kHz (left panel) and (b) absolute threshold at 4 kHz against exposure (right panel). Exposure is plotted on a cube-root scale in both panels. Data are for the 36-member Older group. In both panels, the reduced set used in the ANOVAs is plotted with black symbols. Dots and circles show data for male and female members of the LN sub-group, respectively. Downward and upward pointing triangles show data for male and female members of the HN sub-group respectively. Gray “star” and “plus” symbols show data for male and female members of the full data set, respectively. **r**_**30**_ and **r**_**34**_ in the upper right-hand corner of each panel denote the correlations for the remaining members of the ANOVA data set or the full data set, respectively, after removal of participants with zero exposure.

For the Older group, there was a significant correlation between exposure and AM detection threshold at 10 dB SL for the frequency of 3 kHz. This is illustrated in the left panel of Fig. 9. AM detection tended to worsen with increasing exposure. This is consistent with the pattern of results shown in the left panel of Fig. 5. There was a marginally significant negative correlation between absolute threshold at 4 kHz and exposure, as illustrated in the right panel of Fig. 9. The absolute threshold tended to be lower (better) in those with higher noise exposure. This paradox will be addressed later. No such correlation was found at either 3 or 6 kHz. There were significant negative correlations between DPOAE level and absolute threshold at 4 and 6 kHz (**r**_**36**_ = −0.351, *p* = 0.036 and **r**_**36**_ = −0.335, *p* = 0.046, respectively).

For the Young and Older data sets combined, the only significant correlation involving self-report of recreational noise exposure was with HL6k: **r**_**72**_ = 0.249, *p* = 0.035 (Data for seven subjects were excluded due to zero-valued exposure).

Correlation tables for the full-member data sets (thereby precluding correlations with noise exposure), are given in Table 4, section (1) for the Young group, section (2) for the Older group and section (3) for the Young and Older groups combined. Only with the last data set did DPOAE levels correlate significantly with measures of Absolute threshold at the same frequency, for all three frequencies tested. As would be expected, there were also strong correlations between a given measure across different frequencies. While Absolute thresholds at 3 and 4 kHz were correlated with several measures, Absolute thresholds at 6 kHz were correlated only with DPOAE levels at the same frequency and Absolute thresholds at 4 kHz.

## 4 Discussion

### 4.1 Interpretation of the pattern of results

As described in the introduction, substantial loss of function of IHCs can occur with minimal effect on absolute thresholds. Therefore, associations between noise exposure and absolute threshold can reasonably be attributed mainly to OHC dysfunction. OHC dysfunction is also expected to be associated with reduced DPOAE levels. OHC dysfunction in isolation may lead to a recruitment-like effect that improves AM detection. On the other hand, dysfunction of IHCs may lead to reduced fidelity of coding in the auditory periphery and to impaired AM detection. These points are used to guide interpretation in the following discussion.

#### 4.1.1 Young group

The results for the Young group suggest that noise exposure was associated with OHC damage, shown as:

(1) Elevated hearing threshold at 6 kHz for the HN group relative to the LN group, and a correlation between absolute threshold at 6 kHz and the amount of exposure (Fig. 3);
(2) A negative correlation between the level of DPOAEs and absolute threshold, but only at 4 kHz (Table 4(1)).

The results for the Young group showed poorer AM detection at 10 dB SL for the male HN sub-group relative to the male LN sub-group. This occurred even for frequencies where absolute thresholds and DPOAE levels did not differ for the LN and HN groups. This poorer AM detection may reflect an effect of noise exposure on IHC function. The reason that the effect was only apparent for the males may be connected with the finding that men tend to prefer higher levels than women when listening to PMPs, and perhaps in other situations. This observation lay behind the choice of Vinay and Moore (2010) to include only male participants in their groups. It is also interesting to note that, although the control group of Stone et al. (2008) was gender-balanced, their exposed group comprised five males whose predominant source of exposure was performance at rock concerts, while the sole female mainly attended nightclubs, for which exposures are about 5 dB lower in level but of longer duration than for rock concerts.

In both the present study and that of Stone et al. (2008), adverse effects of noise exposure on envelope detection/discrimination occurred only for low SLs. It is possible that relatively poor AM detection and discrimination occur only when peripheral information is highly impoverished, and this requires both IHC dysfunction and stimuli evoking a restricted excitation pattern.

#### 4.1.2 Older group

The Older group also showed signs of noise-induced OHC dysfunction, indicated by decreasing level of DPOAEs with increasing absolute threshold at 3 and at 4 kHz. In addition they showed improving AM detection with increasing absolute threshold at both 3 and 4 kHz, indicative of a recruitment-like process (Fig. 8, both panels).

As for the Young group, there was also evidence for IHC dysfunction related to noise exposure. AM detection at 10 dB SL was worse for the HN than for the LN sub-group, at 3 and 4 kHz only (Fig. 5, both panels). Also AM detection thresholds increased with increasing exposure, but only at 3 kHz (Fig. 9, left panel).

For the Older group there was a marginally significant negative correlation between noise exposure and absolute threshold at 4 kHz (Fig. 9, right panel), but not at 3 or 6 kHz. This might reflect a type 1 error, or it might be a consequence of the fact that at least one participant had a low calculated exposure, since she was not a habitual attendee at high-noise events, but, by self report, had a previous history of long-term use of PMPs at high levels. Since she no longer used such devices, it was not possible to measure a typical listening level, so it was not possible to adjust her exposure values to reflect this. This did not appear in the self-reports from any other participants. Removal of her data point made the correlation non-significant.

It appears counter-intuitive that AM detection should improve with increasing absolute threshold but worsen with increasing noise exposure. However, these findings make sense if, as suggested earlier, the absolute thresholds in the Older group were mainly determined by OHC function; worsening OHC function leads to higher absolute thresholds but improved AM detection because of a recruitment-like effect. On the other hand, high noise exposure can lead to IHC dysfunction, which leads to poorer AM detection with little or no effect on absolute thresholds, at least for the exposure durations tested here.

#### 4.1.3 Combination of data for Young and Older groups

The ANOVAs based on the combined data sets, as well as the correlation analyses, give a somewhat clearer picture. There was a series of effects associated with OHC function:

(a) Absolute threshold at 6 kHz was higher for the HN than for the LN sub-groups (right panel of Fig. 6).
(b) The Older group had higher absolute thresholds than the Young group, especially at 3 kHz (left panel of Fig. 6). This may have been due to ageing per se, to greater overall noise exposure in the Older group, or to other environmental factors.
(c) Increasing absolute thresholds at 3, 4 and 6 kHz were associated with decreased levels of DPOAEs (Table 4(3)). The effect was weakest at 3 kHz.
(d) The absolute threshold was higher for the Older than for the Young group, and, for the former, AM detection was better for the LN than for the HN sub-groups (Table 3(1) and left-hand panel of Fig. 5).

Poorer AM detection for the HN than for the LN group, perhaps indicative of IHC dysfunction, was found at 10 dB SL for the combined data for the Young and Older groups (Fig. 7, right). However, the Older group actually showed better AM detection than the Young group at 10 dB SL (Fig. 7, left).

Vinay and Moore (2010) found better AM detection for a noise-exposed group (habitual users of PMPs) than for a control group, but only for a 6-kHz carrier frequency, and not for carrier frequencies of 3 and 4 kHz. The finding of better AM detection for the noise-exposed group seems discrepant with our findings. A possible reason for the discrepancy is that use of PMPs generally results in lower exposure levels than attendance at clubs or live concerts. It may be the case that moderate exposure levels produce mainly OHC dysfunction, leading to improved AM detection, while higher exposure levels produce IHC dysfunction in addition, leading to poorer AM detection.

The better AM detection for the Older than for the Young group at 10 dB SL (Fig. 7, left) might have been influenced by the fact that the Older group contained more males than females in the HN sub-group and more females than males in the LN group.

### 4.2 Possible links with data from animals

For the Young group, a difference in absolute threshold between the LN and HN sub-groups, probably indicating OHC dysfunction, occurred only at 6 kHz (Fig. 3). However, poorer AM detection in the male HN sub-group, probably indicating IHC dysfunction occurred for all frequencies, suggesting a possible difference in the relative effects of noise exposure as a function of cochlear position. The pattern was also found for the combined data of the Young and Older groups.

In cats, the relative loss of stereocilia on OHCs and IHCs varies according to characteristic frequency (CF) within the cochlea relative to the center frequency of the narrow-band noise exposure (Liberman and Dodds, 1984). This is most apparent in panel C in each of Fig. 3, Fig. 4 of Liberman and Dodds. For some CFs, there can be significant damage to IHC stereocilia with little damage to OHC sterocilia. More recent work has also demonstrated dissociations between aspects of OHC dysfunction and IHC dysfunction (Kujawa and Liberman, 2009, Lin et al., 2011, Liu et al., 2012). Following noise exposures designed to produce only temporary threshold shifts in mice and guinea pigs, and hence negligible OHC damage, the studies showed a loss of synaptic contacts at the base of IHCs. Note, however, that this loss of synaptic contacts was argued to affect low- and medium-spontaneous-rate fibers (Furman et al., 2013), which are presumed to contribute to perception only for moderate to high-level signals, whereas the effects reported here were obtained at low SLs.

## 5 Summary and conclusions

Absolute thresholds, DPOAEs and AM-detection thresholds were measured for Young and Older participants with variable degrees of exposure to high-noise events (defined as exceeding 95–100 dBA), using test frequencies at which effects of noise exposure are typically first observed. The results can be interpreted in terms of two distinct physiological effects, associated with OHC function on the one hand, and IHC/synaptic/neural function on the other hand.

Higher absolute thresholds were associated with improved AM detection at 3 and 4 kHz, but not at 6 kHz. Ageing, which was accompanied by higher absolute thresholds, was also associated with improved AM detection. The levels of DPOAEs reduced with increasing absolute threshold, at 4 kHz for the Young group, and at 4 and 6 kHz for the Older group. We attribute these effects to impaired OHC function, which reduces the gain of the active mechanism in the cochlea and leads to steeper input–output functions (Robles and Ruggero, 2001).

A second pattern was observed when comparing results for participants in the HN and LN sub-groups. At the lowest SL tested, AM detection was poorer for the HN than for the LN sub-groups, only for the males in the Young group, but for all members in the Older group. Greater exposure was associated with poorer AM detection, but only at 3 kHz. We interpret these results as primarily reflecting IHC dysfunction for a CF of 3 kHz.

It seems clear that noise exposure can produce both OHC dysfunction and IHC dysfunction, the balance between the two depending on the intensity and duration of the exposure and perhaps on frequency. Both OHC- and IHC-related effects were apparent at 3 and 4 kHz, while OHC-related effects were clearest at 6 kHz.

The idea that noise exposure can produce both OHC- and IHC-related effects is consistent with studies using animals. There is, however, at least one discrepancy. It has been proposed that effects of noise on IHC/synaptic/neural function primarily affect the high-threshold, low-spontaneous rate neurons (Lin et al., 2011, Furman et al., 2013). If this were the case for humans, one would expect deleterious effects on AM detection to be observed at high rather than low sound levels. We found adverse effects of noise exposure on AM detection only for the lowest SL used. The apparent discrepancy may reflect differences in the type of exposure. In the studies of Kujawa and Liberman, 2009, Lin et al., 2011 and Liu et al. (2012) the noise exposures were carefully titrated so as to produce only a temporary threshold shift. Our HN sub-groups showed some evidence of permanent threshold shift, perhaps because of the longer durations and higher levels of their exposures. Such exposures may affect the function of high-spontaneous rate neurons in humans, leading to deficits in the peripheral coding of low-level sounds, as observed here.

The difference between our results and those of Vinay and Moore (2010) is consistent with the idea that there is a critical level for humans, and that it lies at the upper end of the range of preferred listening levels available from PMPs but is commonly exceeded at nightclubs and rock concerts.

Our results indicate that noise-related damage to both OHCs and IHCs/synapses/neurons can occur in a very young group (mean age 21 years) and it progresses with continued exposure. However, the primary tool used here, AM detection, appears not to be very sensitive, since OHC and IHC damage produce opposite effects. Also, there was considerable overlap in the AM detection thresholds for the LN and HN sub-groups, for both the Young and Older groups, so AM detection does not appear to be a promising way of identifying early signs of damage from noise exposure in humans.

## Figures and Tables

**Table:** 

Day-to-Day noise exposure		
Have you had a job with high noise exposure? If so, please detail when, where and for how long. Did you use appropriate ear protection?		
Do you have any noisy hobbies, such as motor car racing or shooting? If so, how long have you had these hobbies and how regular are they?		
Do you play a musical instrument(s)? If yes: (1) what instrument(s) (2) since what age ? and (3) how often per week do you play (hrs)		
What genre(s) of music?		

**Personal Music Player (PMP)**		

Average exposure per week (hrs)		
How loud do you listen to your PMP (Out of 10)		
Type of PMP (make and model)		
Type of headphones, i.e. ear buds (basic), in ear, over ear		
Make and model of headphones (if known)		

**Gigging/Clubbing**		

	**Gigging**	**Clubbing**
Have you ever been gigging/clubbing?		
When did you first start gigging and or clubbing? (Age)		
How often did you go as an undergraduate? (Hours per week)		
Most attended Venue(s)		
Since then in 5 year periods what sort of average exposure (hours per week)	Hours per week	Hours per week
Aged 21–25 years		
Aged 26–30 years		
Aged 31–35 years		
Do you wear ear protection? If yes, what sort? E.g. plugs, defenders, etc.		
